# Fluid and water balance: a scoping review for the Nordic Nutrition Recommendations 2023

**DOI:** 10.29219/fnr.v67.9975

**Published:** 2023-11-13

**Authors:** Per Ole Iversen, Mikael Fogelholm

**Affiliations:** 1 Department of Nutrition, Institute of Basic Medical Sciences, University of Oslo, Oslo, Norway; 2 Department of Haematology, Oslo University Hospital, Oslo, Norway; 3 Department of Food and Nutrition, University of Helsinki, Helsinki, Finland

**Keywords:** water, fluids, beverages, hydration, recommendations

## Abstract

Water, being an essential nutrient, is crucial for all life. Adequate maintenance of body water compartments is required for optimal fluid balance, which is a prerequisite for cellular homeostasis and blood pressure regulation. Water balance is the result of dietary intake of both fluids and foods as well as metabolically produced water, excretion from the kidneys and losses from other sources (e.g. sweat and feces). The water content in food items varies considerably and ranges from about 5% in nuts to 90% in many fruits and vegetables. Intake of drinking water and beverages also provides varying amounts of water. In everyday life assessment of water balance (i.e. hydration status) is challenging as clinical observations are inaccurate. There is no data regarding drinking water intake in the Nordic or Baltic countries.

## Popular scientific summary

- Water is an essential nutrient
- The main sources are drinking water and beverages.
- An optimal fluid balance is needed to maintain normal physiological functions and health.
- Urine osmolality is a commonly used measure of hydration status.
- The requirement for fluids varies considerably between individuals and is affected by both physical activity and the ambient climate.

Water is an essential nutrient and thus a source of all life. Human growth, development, survival, and reproduction of life require water. The lack of water undermines survival and threatens existence. Safe water for human consumption is critical for maintaining good health. In addition comes the role of safe water for agriculture and for sanitary (i.e. adequate methods for safe disposal of human waste such as feces and urine) and hygienic (i.e. behaviors that can improve cleanliness) purposes. Efforts to improve these three factors (water, sanitation and hygiene) are often referred to as the WASH initiative (1).

Water is also the source of death. Deadly conflicts over access and control of water have deep historical roots, spanning millennia back in recorded history. Thus importantly, the pivotal role of water is described in several human rights provisions, including the Convention of the Rights of the Child (Article 24) and the International Convention on Economic, Social, and Cultural Rights (elaborated on in General Comment 15), and is highlighted in Voluntary Guideline 8c on the Right to Adequate Food as adopted by the Food Agricultural Organization. In short: The human right to water is indispensable for living a life in dignity.

United Nations Sustainable Development Goal no. 6 ‘Clean water and sanitation’ also highlights the importance of safe water.

In this review, we focus on the importance of human fluid consumption with emphasis on giving updated, evidence-based dietary reference values for drinking water intake (Box 1).

Box 1. *Background papers for Nordic Nutrition Recommendations 2023*

- This article is one of many scoping reviews commissioned as part of the Nordic Nutrition Recommendations 2023 (NNR2023) project (3).
- The articles are included in the extended NNR2023 report but, for transparency, these scoping reviews are also published in Food and Nutrition Research.
- The scoping reviews have been peer reviewed by independent experts in the research field according to the standard procedures of the journal.
- The scoping reviews have also been subjected to public consultations (see report to be published by the NNR2023 project).
- The NNR2023 committee has served as the editorial board.
- While these articles are a main fundament, the NNR2023 committee has the sole responsibility for setting dietary reference values in the NNR2023 project.

## Methods

This review is an update of the NNR2012 recommendations (2), and has been prepared according to the protocol developed by the NNR2023 project (3). The sources of evidence for this review follow the eligibility criteria described by Christensen et al. (4). A separate literature search did not identify any qualified systematic reviews.

The following literature search was conducted in the PubMed database:

1. We first identified systematic reviews linking water intake to human health outcomes using this search string: (balance, water electrolyte[MeSH Terms] OR “water balance”[Title/Abstract] OR “fluid balance” [Title/Abstract] OR “hydration”[Title/Abstract] OR “water intake”[Title/Abstract]) AND (“2011”[Date - Publication] : “3000”[Date - Publication]) AND Humans[Filter] AND Systematic Review[Publication Type]. This search was performed on August 18, 2021 and identified 195 reviews.
2. On August 18, 2021 we also performed a similar search as above, exchanging the term “Systematic Review” with “Randomized Trial”, yielding 92 studies.
3. To identify studies on how to assess hydration status, we used this search string on August 18, 2021: (balance, water electrolyte[MeSH Terms] OR “water balance”[Title/Abstract] OR “fluid balance”[Title/Abstract] OR “hydration” [Title/Abstract] OR “water intake”[Title/Abstract]) AND (“2011”[Date - Publication] : “3000”[Date - Publication]) AND Humans[Filter] AND hydration status AND assessment, which identified 10 studies.
4. In an additional search conducted on August 18, 2021, we had a broader approach by using the same search string as under aforementioned points 1 and 2, but without specifying study type/design.
5. To identify data on drinking water consumption, we did a PubMed search on September 12, 2021, using the search string: (water[title/abstract] OR fluid[title/abstract]) AND intake[title/abstract] AND (Finland[title/abstract] OR Sweden[title/abstract] OR Norway[title/abstract] OR Denmark[title/abstract] OR Iceland[title/abstract] OR Estonia[title/abstract] OR Latvia[title/abstract] OR Lithuania[title/abstract; which identified 215 studies in total and 96 for the last 10 years.

For comparisons we included references to guidelines of water intake from other authoritative bodies (i.e. National Academy of Sciences, Engineering, and Medicine [NASEM] in the US and the European Food Safety Authority [EFSA]). In addition, we also scrutinized the reference list of some of the studies retrieved in our literature searches.

## Physiology: Regulation of water balance

### Body water content

Total body water (TBW) comprises approximately 50–65% of a person’s body weight (5, 6). Muscle mass contains 70–75% water, while water in fat tissue typically constitutes between 10 and 20%. In newborns, who have developed in an aqueous milieu, body water content at birth is approximately 75% of body mass (7). The infant’s relative water content decreases rapidly throughout the first year of life to about 60% and remains relatively stable throughout childhood until adolescence. The average TBW is lower in adult women compared with men due to higher relative fat mass. Approximately two-thirds of TBW is confined to the intracellular compartment and the remaining third is located extracellularly. In the extracellular compartment, about 75% of the water is in the interstitium and 25% is a component of blood plasma.

### Sources of water intake

Solid foods provide an average of 600–800 mL of water per day (8). The water content in food items varies considerably and ranges from about 5% in nuts to 90% in many fruits and vegetables. Intake of fluids, that is, drinking water and beverages, also provides approximately 700 to 1,400 mL/day of water. For example, in Sweden, daily drinking water consumption is 6–7 glasses (9), in total about 1,200 to 1,400 mL. Fluid water intake generally accounts for ~ 70–80% of total water consumed, and ~20–30% of total water intake comes from solid foods (10). Finally, the oxidation of fat, carbohydrates, and protein yields an additional 300 to 350 mL of water per day. In addition, water also comes from other metabolic processes.

### Water balance and functions

Water balance (i.e. water input vs. output) is influenced by dietary intake, physical activity level, age, and environmental conditions (6). The brain actively regulates both TBW volume (within 0.5% day-to-day variation) and blood concentration (within a normal plasma osmolality range of 285–295 mOsm/kg), across a wide range of total water intake (11). There are wide within- and between-individual variations in daily water needs, hence it is difficult to make other than average and general estimations. Body mass is an important determinant – large body mass (particularly muscle mass) leads to higher total water requirements (11).

Water is a crucial nutrient and euhydration is necessary for optimal daily functioning (11). Water is needed to maintain normal physiological functions (e.g. blood pressure, pH, internal body temperature) and health, and to transport essential substances (e.g. oxygen, carbon dioxide, water, and glucose) to and from cells, regulate body temperature, lubricate joints, provide structure to cells and tissues, and to help preserve cardiovascular function (6). Water deficits can impair physical performance (12, 13) and recent research suggests that cognitive performance may also be impacted (6). The progressive maturation of kidney function by around the age of 2 years as well as a higher body surface-to-body mass ratio, translates into higher insensible water loss through the skin. This explains in part why children have higher water requirements relative to their body mass when compared with adults (14). The skin, kidneys, lungs, and digestive system are all sources of water output. Respiratory water loss averages 250–350 mL/day in sedentary adults (6). On average insensible water losses are about 450 mL/day; however, during vigorous physical activity in a hot environment, losses in excess of 2,000 mL/h are possible (13). Urine output generally ranges between 1,000 and 2,000 mL/day. Total water output is approximately 1,500–3,100 mL/day for adults in temperate climates (6).

### Regulation of water balance

The regulation of water balance is based on a feedback mechanism involving the hypothalamus, the neurohypophysis, and the kidneys (15). Arginine vasopressin (AVP), also denoted anti-diuretic hormone, is the body’s primary water-regulating hormone. It functions to maintain body water balance by keeping plasma osmolality within narrow limits and allowing the kidneys to alter water excretion in response to the body’s needs, in conjunction with thirst.

Osmoreceptors in the hypothalamus sense plasma osmolality (15). When water loss exceeds intake, blood volume decreases and plasma osmolality increases. An increase in osmolality above a physiologic threshold (290 to 295 mOsm per kilogram of water in most persons) leads to an increased secretion of AVP that binds to the vasopressin 2(V2)-receptors in the kidney. This increases the permeability to water in the last portion of the nephron, leading to a reabsorption of solute-free water and consequently a decreased water output in urine (16, 17). An increase in the osmolality also activates the renin- angiotensin system, leading to increases in renin and angiotensin II concentrations (15). The latter, along with aldosterone, promote sodium and chloride reabsorption in the kidneys and thus water via osmosis, and thereby decreases urine output. Increased renal reabsorption of water in response to AVP lowers plasma osmolality, thereby reducing the stimulus for AVP secretion and thirst and completing the feedback loop (15). The physiology is reversed in the case of an excess extracellular fluid in the body: osmolality decreases, leading to decreased renal water reabsorption and excretion of diluted urine.

### Thirst

Water intake and fluid balance are regulated by physiological (e.g. osmoreceptors in the brain and mouth) and non-regulated (e.g. social, cultural, behavioral) factors (6). Thirst is the primary means by which humans sense dehydration. The thirst sensation is triggered with a body water loss of 1–2%. Several factors influence the onset of thirst, including blood pressure, blood volume, AVP, and angiotensin II (18). The primary stimulus for thirst, however, is serum osmolality. Older adults (>65 years) experience reduced thirst and water intake, reduced maximal renal concentrating ability, higher plasma AVP concentration during water restriction, and reduced ability to excrete a water load when compared with younger adults (19). When fluid is consumed, the deceased osmolality is sensed by the osmoreceptors, leading to reduced AVP (15) and restoration of urine output. Moreover, the thirst sensation fades when the serum osmolality decreases and the blood volume increases (20, 21).

## Assessment of hydration status

Based on the literature search, we found that two methods are usually applied regarding objective assessments of hydration status. In healthy individuals, bioelectrical impedance data on body water compartments agree favorably with the deuterium dilution (often considered the ‘gold standard’) (22). However, the bioelectrical impedance method is subject to variations among individuals and among manufacturers of the equipment. The other commonly used method is based on urine osmolality and apparently a spot urine sample is sufficient and can be used instead of urine sampled over 24 h (23). NASEM concluded that urine volume, color and osmolality are often used, but not very accurate indicators of hydration status (24). Changes in body weight may also give a rough indication of changes in body water content (and hydration status) if other factors are carefully controlled (24). Notably, clinical signs of dehydration (dried mucous membranes) or overhydration (e.g. edema) are too unreliable for quantification and scientific purposes. The choice of hydration assessment method and its interpretation is dependent on the type of dehydration. For example, iso-osmotic hypovolemia does not increase plasma or serum osmolality due to the concurrent loss of salt and water (25).

## Intake of drinking water in the Nordic and Baltic countries

Our literature search did not reveal any relevant data on drinking water intake in the Nordic or Baltic countries. However, two Nordic studies can be mentioned. Save-Soderbergh et al. (9) studied the consumption of tap water by traditional (telephone interview, web questionnaire) and novel (SMS) methods in Sweden (9). Based on the results from the SMS study, the authors suggested using 1 bv bvb mL/day for the average adult population and 2.5 L/day for high consumers for risk assessment of cold tap water consumption. As 46% of the tap water consumed is heated, this study suggested using 1.85 L/day for total tap water. It should be noted, however, that this is not the same as total fluid consumption. A Norwegian study showed that 90% of the participants (14–15 year-old school students in one county) met the recommended daily water intake of at least 1 glass – a recommendation which in fact may be considered rather low (26).

## Health outcomes relevant for Nordic and Baltic countries

Euhydration and an adequate water intake are necessary for a wide array of normal body functions, including physical and cognitive performance (6, 10, 11, 25). Moreover, there are data from both animal and human studies at least suggesting that even mild hypohydration, in connection to increased plasma osmolality and AVP concentration, may have a deleterious effect on vascular function and thereby this condition may contribute to the development of cardiovascular disease (27).

The data on water intake in Nordic and Baltic countries is lacking, but no indications of a general deficit has been reported (9). It is likely that the most vulnerable population group are older adults (27), hence giving practical advice (e.g. having a bottle filled with water in the fridge) in ensuring adequate fluid intake for this group is important. However, it is possible that a more representative analysis of biomarkers of hydration status could give more insight. In a narrative review, Liska et al. noticed that according to the analysis of combined urine osmolality data from the NHANES 2009–2010 and 2011–2012 surveys, about 1/3 (32.6%) of adults (ages 18–64 years old) and more than half (54.5%) of children and adolescents (ages 6–19 years old) in the US seems to be inadequately hydrated (25).

Other health outcomes relevant for fluid intake are most likely caused by the nutritional content of fluids, rather than the volume of fluid *per se*. The most obvious nutritional concerns are sugar and alcohol content of the drinks. Another example is high saturated (dairy) fatty acid content of, for example, coffee drinks. The health issues of nutrients in fluids are discussed in the NNR2023 scoping reviews related to the specific nutrients.

## Requirement and recommended intakes

Large between- and within-subject variances make it difficult to determine a water requirement for all persons within a life stage (11). For example, the 24-h human water requirement varies with anthropomorphic characteristics, especially body mass. Large individuals require a greater daily total water intake than small individuals. Most healthy people meet their daily hydration needs by letting thirst be their guide. It is virtually impossible to give exact recommendations on daily water intake for healthy subjects because the requirement for fluids shows considerable inter-individual variations, and it is confounded by physical activity patterns and the ambient climate (e.g. temperature and humidity).

NASEM found that the evidence is insufficient to establish water intake recommendations as a means to reduce the risk of chronic diseases (28). Instead NASEM has made general recommendations for adequate intake (AI, cut-off values to prevent deleterious, primarily acute effects of dehydration and to ensure optimal body functions in relation to hydration status) of approximately 2.7 and 3.7 L from all beverages and foods daily for women and men (aged 19 to 30 years), respectively, but has not set an upper level for total water intake. Moreover, NASEM set the AI for total water to 1.3 L per day for children 1–3 years old, 1.7 L per day for children 4–8 years old, 2.4 and 2.1 L per day for 9–13-year-old boys and girls, respectively, and 3.3 and 2.3 L per day for 14–18-year-old boys and girls, respectively (28).

The EFSA set the AI for total water between 2.0 and 2.5 L per day for adult women and men, respectively (24). The AI for total water per day was set to 0.8–1.0, 1.1–1.2, and 1.3, and 1.6 L per day for children aged 0.5–1, 1–2, 2–3, and 4–8 years, respectively. Furthermore, the daily AI for 9–13-year-olds was set to 2.1 L for boys and 1.9 L for girls. The recommended AI for children aged 14 years and older is similar to that of adults. EFSA also recommends an additional 0.3 L of water per day for pregnant women. Moreover, EFSA recommends that lactating women have the same daily AI of total water as non-lactating women plus an extra 0.7 L. For elderly people whose capacity to concentrate the urine is limited and who often have impaired feelings of thirst, a broader safety margin might be needed. The EFSA, however, does not recommend a specific AI for total water intake among the elderly.

The NNR2012 recommendations provided guiding values for daily intake of water and fluids, in addition to water derived from foods (2). Specifically, these guiding values were set to 1–1.5 L daily for adults and children (>14 years) and 1 L for children aged 2–13 years.

## Conflict of interest and funding

The authors declare no conflict of interest.

## Authors’ contributions

POI collected and analyzed the data and drafted the first version of the manuscript. MF collected and analyzed the data and critically reviewed the manuscript. Both authors read and approved the final version of the manuscript.
